# Creep level qualitative evaluating of crushed rock based on uncertainty measurement theory and hierarchical analysis

**DOI:** 10.1038/s41598-024-65222-x

**Published:** 2024-06-22

**Authors:** Shiwei Wu, Qi Mou, Tao Yang

**Affiliations:** 1 Department of Civil Engineering, Southwest Jiaotong University, Chengdu, China; 2 School of Earth Science and Environmental Engineering, Southwest Jiaotong University, Chengdu, China; 3 Sichuan Tongchuan Engineering Technology Development Co., Chengdu, China; 4 Sichuan Highway Planning, Survey and Design Institute Co., Chengdu, China

**Keywords:** Uncertainty measurement theory, Hierarchical analysis, Crushed rock, Creep, Qualitative evaluation, Civil engineering, Computational methods

## Abstract

A large number of tectonically mixed rock belts and complex tectonic zones are distributed in the southwestern part of China. In these areas, high geostress and tectonic stresses have caused some underground rock layers to be crushed and broken, eventually forming crushed rock zones. Which may undergo creep deformation under long-term loads. The manuscript is based on a typical crushed rock in the southwestern China. Firstly, the factors affecting creep deformation were analysed, and the response law of each influencing factor to rock creep is demonstrated. Then, the theory of uncorroborated measures and hierarchical analysis were used to systematically correlate the factors influencing creep. Thereby, a creep level qualitative evaluating model of crushed rock is established. Finally, this model was used to qualitatively evaluate the creep level of the crushed rock in the study area. It is concluded that the creep level qualitative evaluating of this crushed rock is rated as Class II, which is characterised by a low creep level and small creep deformations (0–10 mm). The research results can provide a reference for the creep analysis of crushed rock and provide a basis for the safe construction of engineering slopes.

## Introduction

**Figure 1:**
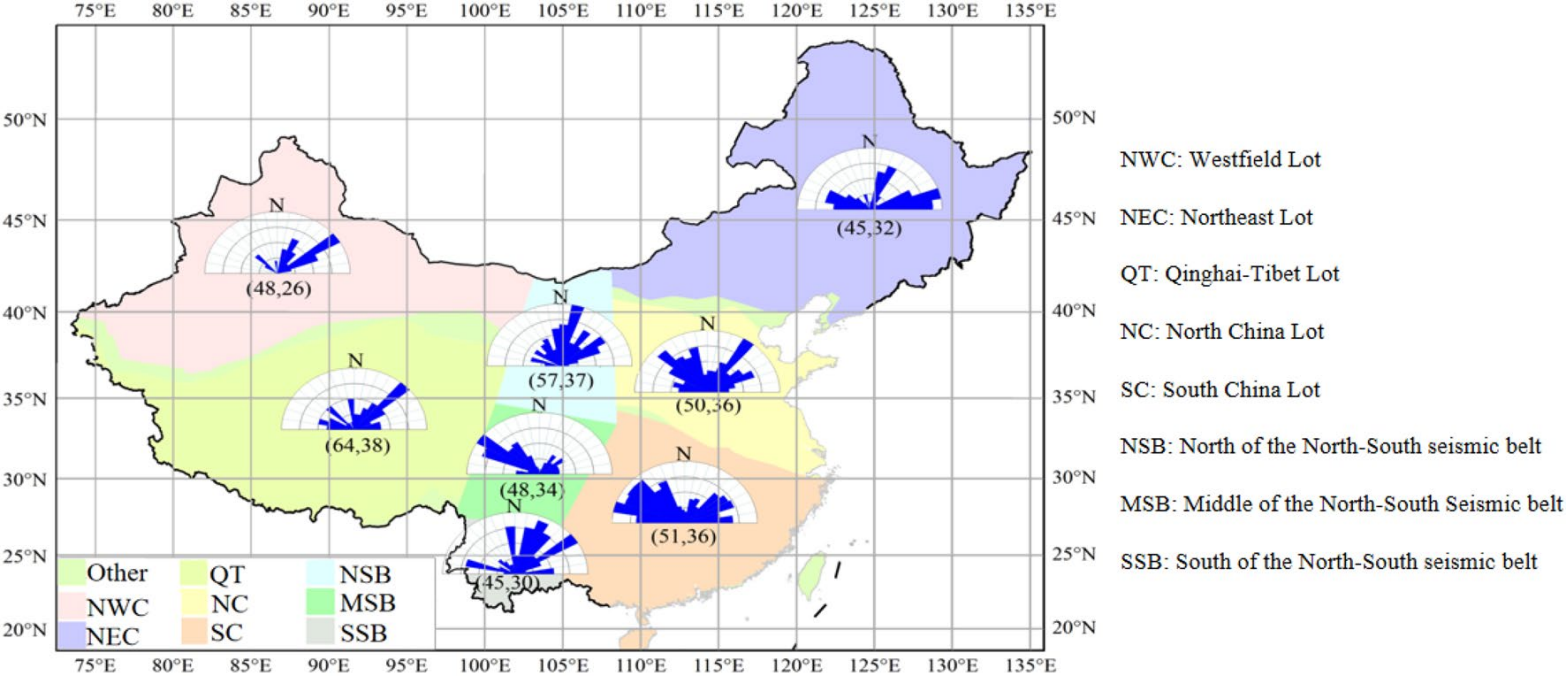
Horizontal stress values and orientation characteristics by region in China.

In the southwest of China lies the world's highest average altitude plateau, the Tibetan Plateau. The Tibetan Plateau was formed as a result of strong tectonic movements between plates, and this process of movement is not done all at once, but continues over a long period of time to this day and beyond. The maximum horizontal stress azimuthal rosette for each region of China is shown in Fig. 1. It can also be seen from the figure that the maximum horizontal geostress in the Qinghai-Tibet Lot is 64 MPa and the minimum horizontal geostress is 38 MPa (buried depth of 2000 m), which is significantly higher than that of other regions in China^1^.

Macroscopically analyse, tectonic movements have resulted in the existence of a large number of tectonic fracture zones in the south-western part of China, and formation of numerous mountains, rivers and canyons. Microscopic analysis, the development of geological tectonics and the high altitude of the mountains in the region are very likely to cause the rock inside the mountains to be in a high stress and complexity environment, resulting in the development of structural planes in the rock and the rock are more fragmented. Resulting in rock development of structural planes and fragmentation.

The paper takes the crushed rock in southwest China as the research object, the geological environment in this region is extremely complex, under the action of long-term high stress environment, and so on, the crushed rock inside the mountain is susceptible to creep deformation. The paper firstly analyses the main influencing factors and influencing mechanisms of rock creep. Then, the theory of computation of the unconfirmed measurement model and the hierarchical analysis method were used to establish a creep level qualitative evaluation model of the crushed rock. Finally, this model was used to qualitatively evaluate the creep level of the crushed rock in the southwestern China. The research results can provide a reference for the creep analysis of crushed rock and provide a basis for the safe construction of engineering slopes.

### Analysis of factors influencing the creep behaviour of rock

#### The effect of lithology on rock creep

**Figure 2:**
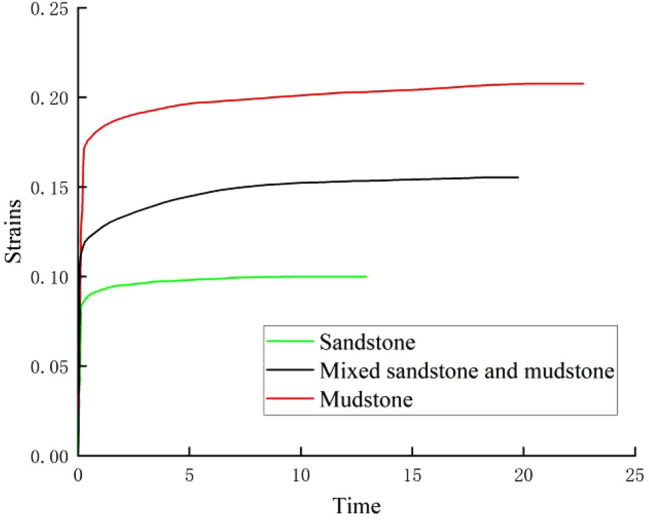
Creep test curves of sandstone, mudstone and mixed sand and mudstone (axial strains).

The influence of lithology on rock creep is mainly manifested in the fact that different rocks have different strength characteristics. In order to study the effect of different lithology on the creep characteristics of rock, Wu Zhiyong^2^ designed a creep test programme for different types of rock mass under the same conditions of stress, temperature, and so on. Creep tests were carried out on sandstone, mudstone, and mixed sand-mudstone respectively, as a means of investigating the response degree to creep of rocks with different softness and strengths. The test results are shown in Fig. 2. As can be seen from Fig. 2, the creep deformation size of the three rock types: sandstone < mixed sand-mudstone < mudstone. It can be seen that, all other things being equal, a rock with softer lithology has a more pronounced creep phenomenon and greater creep deformation compared to a rock with harder lithology.

#### The effect of rock structure on rock creep

The effect of rock structure on rock creep is manifested in two main ways. Firstly, the degree of fragmentation of the rock. For the more complete rock, the less distribution of structural planes and cracks, therefore, its resistance to creep deformation will be stronger, the less likely to creep. On the contrary, for the more broken rock, the more distribution of structural planes and cracks, significant creep deformation of the rock occurs under the long-term external forces. The code of Practice for Geotechnical Investigation classifies rocks with varying degrees of fragmentation into five categories^3^ (see Table 1 for details).

**Table 1:** Classification of rock mass integrity.

Integrity	Complete	Relatively omplete	Relatively crushed	Crushed	Extremely crushed
Integrity index	> 0.75	0.75–0.55	0.55–0.35	0.35–0.15	< 0.15

Secondly, the properties of the structural planes of the rock also have a great influence on rock creep. For rocks with rigid structural planes (structural planes with high friction coefficient, mostly without fillers, small openings), their properties are relatively stable and not susceptible to creep. Nevertheless, for rocks with weak structural planes (structural planes with low friction coefficient, mostly clay filled, wide openings), their ability to withstand creep is weak and they are susceptible to creep deformation^4^.

#### The effect of stress environments on rock creep

**Figure 3:**
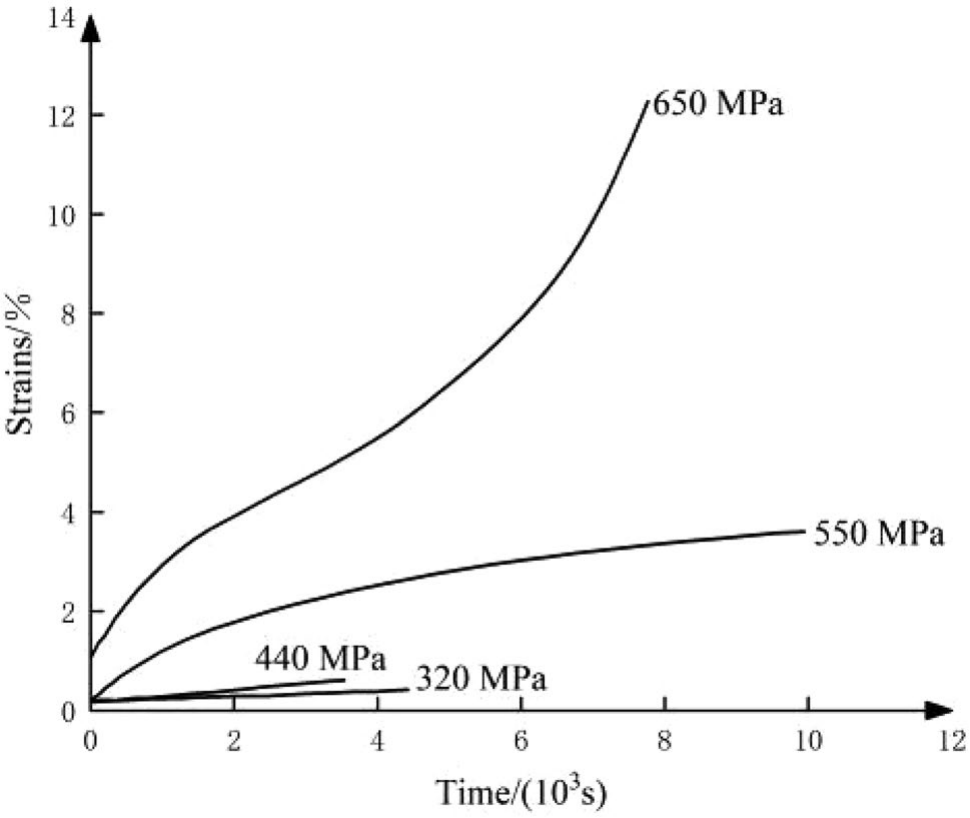
Creep curves of solnhofen limestone under different axial pressures.

The effect of the stress environments on rock creep is mainly reflected in both axial pressure and confining pressure. Firstly, for the axial pressure aspect, Griggs designed a scheme for creep experiments on solnhofen limestone specimens using different axial pressure at 500 MPa confining pressure, the results are shown in Fig. 3. It can be found from the figure, when the confining pressure is certain, the axial pressure is slightly greater than the peripheral pressure (such as 650 MPa), three stages of typical creep deformation can then be seen. When the axial pressure is small, only the first two creep stages can occur. It can be seen that, all other things being equal, the higher the axial pressure, the more intense the creep occurs, and conversely, the lower the axial pressure, the weaker the creep phenomenon of the rock^5^.

**Figure 4:**
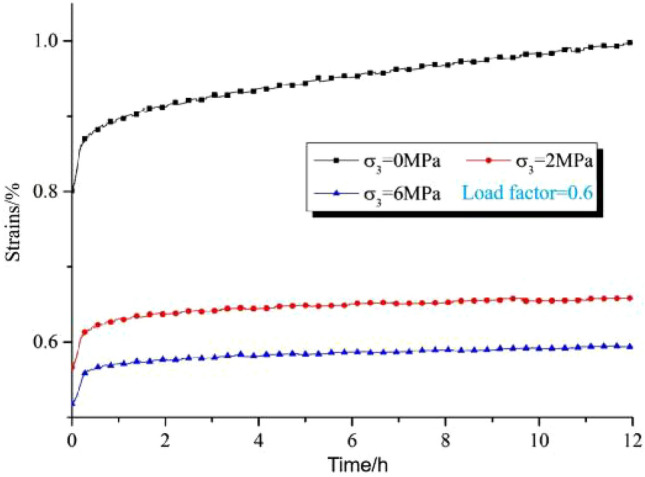
Creep curves of sandstones at different confining pressures.

In order to study the effect of different confining pressures on the creep characteristics of the rock, Wei Yao^6^ designed a creep deformation test of the rock with different confining pressures under the same conditions of lithology, temperature, and so on. The test results are shown in Fig. 4, from the figure, it can be found that under the same conditions, the higher the confining pressure, the rock can show lower creep deformation characteristics (Load factor = 0.6 means that the axial stress remains unchanged during loading and the size is 0.6 times the peak axial strength in conventional triaxial compression).

#### The effect of temperature on rock creep

**Figure 5:**
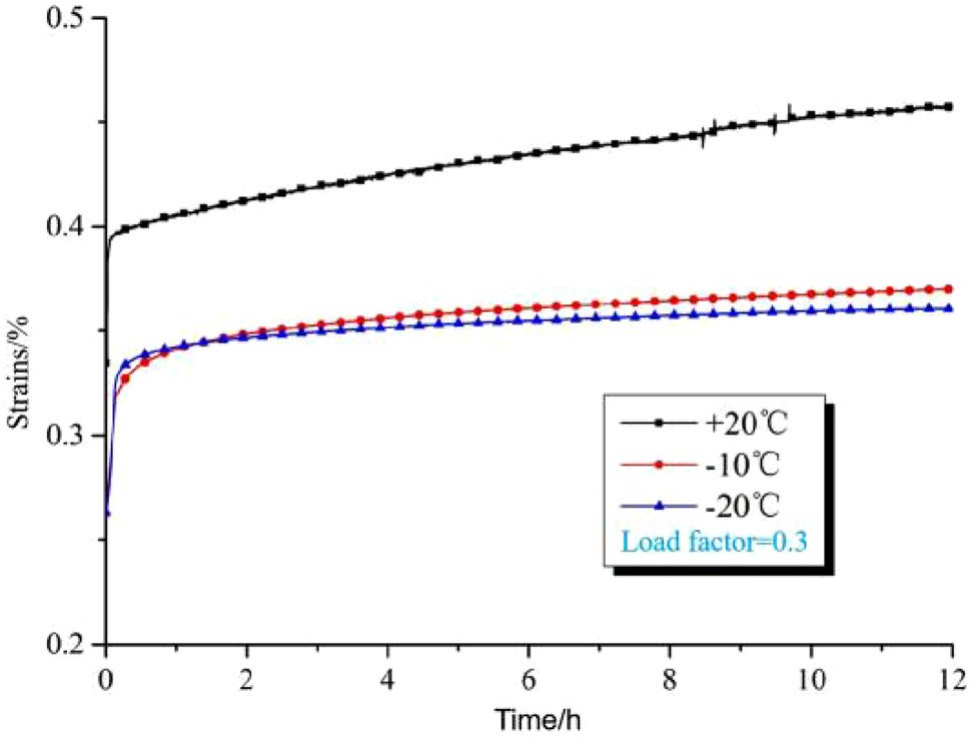
Creep curves of sandstone at different temperatures.

In order to study the effect of different temperatures on the creep characteristics of the rock, Wei Yao^6^ designed a creep deformation test of the rock with different temperatures under the same conditions of lithology, stress, and so on. The test results are shown in Fig. 5, from the figure, it can be found that under the same conditions, the higher the temperature, the more rapid the development of the creep of the rock specimen, the macroscopic manifestation of creep deformation is larger; Meanwhile the lower the temperature, the rock specimen creep development is slower, the macroscopic manifestation of creep deformation is smaller (Load factor = 0.3 means that the axial stress remains unchanged during loading and the size is 0.3 times the peak axial strength in conventional triaxial compression)^7^.

#### The effect of humidity on rock creep

**Figure 6:**
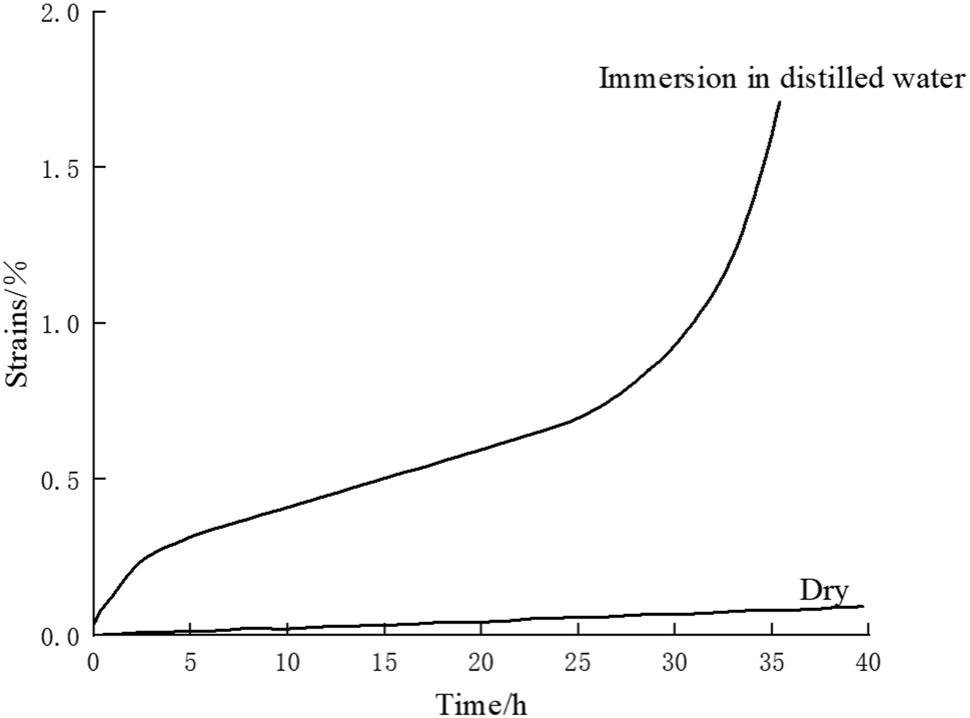
Creep curves of snowflake gypsum at different humidity.

Griggs conducted uniaxial creep tests by immersing snowflake gypsum in different solutions as a means of investigating the pattern of humidity effects on rock creep, and the results are shown in Fig. 6. As can be seen from the figure, the creep curve of the snowflake gypsum changed very significantly after immersing it in the solution compared to the dry condition, and the creep deformation increased greatly. Therefore, it can be seen that under the same conditions of stress and temperature, and so on, the creep deformation of the rock in solution is greater than that under dry conditions.

For rock in natural slopes, the influence of the creeping behaviour of the rock is mainly due to natural environmental effects, such as lithology, rock structure, axial pressure, confining pressure, temperature, humidity, and so on. As for the rock in the engineered slopes, in addition to considering the action of the natural environment, but also to consider the impact of construction, such as excavation, filling, vehicle loads and other operations will cause changes in the stress environment of the rock, which will lead to changes in the creep behaviour. Therefore, the study of rock creep should be carried out depending on the overall environment to which the rock is subjected.

The manuscript summarises a large number of theoretical studies and test results on rock creep at home and abroad, and analyses the factors affecting the creep behaviour of rock, including lithology, rock structure, axial pressure, peripheral pressure, temperature, humidity, construction and so on^8^. The response law between rock creep and various influencing factors is explored too. However, in the case of real engineering can not exist only a single factor, the creep of the rock is bound to be affected by the coupling of various factors, only a single factor is obviously unreasonable. So it is necessary to find a way to carry out a multi-factor analysis of the creep of the rock.

### Evaluation methods and steps

#### Determining the computational matrix based on the theory of uncorroborated measures

Suppose that the object A to be evaluated has n impact factors^9,10^ a_1_, a_2_, …, a_n_, then the object can be written as A = {a_1_, a_2_, …, a_n_}. And for each impact factor a_i_ (i = 1, 2, …, n) there are P evaluation levels e_1_, e_2_, …, e_p_, and all have e_1_ > e_2_ > ⋯ > e_p_, noting that P = {e_1_, e_2_, …, e_p_}^11,12^.

Firstly, each impact factor of the evaluation object is rated according to the enterprise scoring method or inductive method^13,14^ and ${\sum }_{j=1}^{p}{a}_{ij}=100$. Where a_ij_ denotes the observed value of indicator a_i_ at the j-th evaluation level e_j_ (j = 1, 2, …, p), Normalising the resulting observations to obtain u_ij_ = a_ij_/100 denotes the uncorroborated measure of influence factor a_i_^15,16^, count u_ij_ = {a_i1_, a_i2_, …, a_ip_} (i = 1, 2, …, n), u_ij_ is then the uncorroborated measurement matrix as shown in Eq. (1).

1 $$\mu_{ij} = \left( {\begin{array}{*{20}c} {\mu_{11} } & {\quad \mu_{12} } & {\quad \ldots } & {\quad \mu_{1p} } \\ {\mu_{21} } & {\quad \mu_{22} } & {\quad \ldots } & {\quad \mu_{2p} } \\ \ldots & {\quad \ldots } & {\quad \ldots } & {\quad \ldots } \\ {\mu_{n1} } & {\quad \mu_{n2} } & {\quad \ldots } & {\quad \mu_{np} } \\ \end{array} } \right)$$

#### Determining the indicator weights based on hierarchical analysis

**Figure 7:**
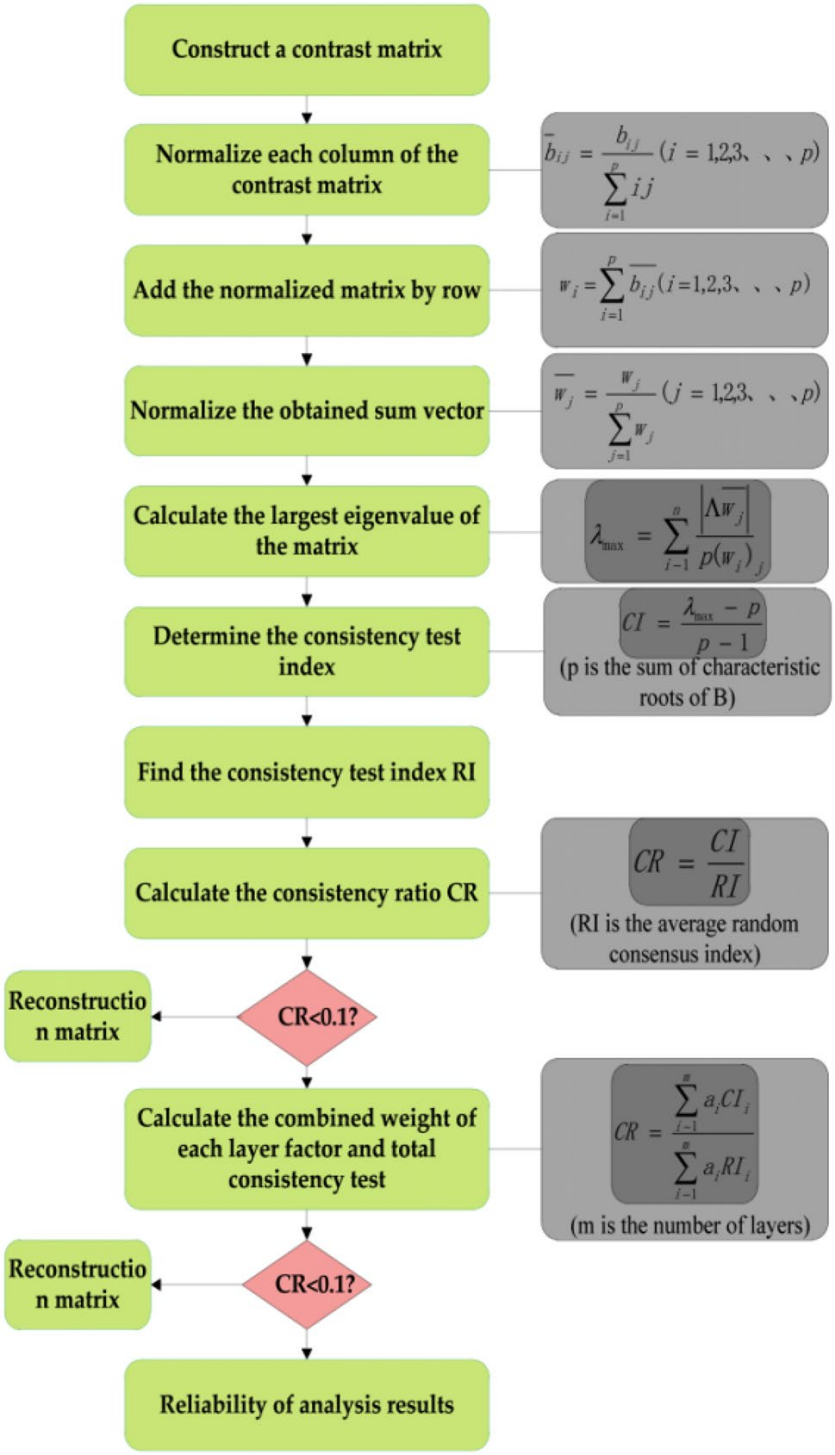
AHP weight calculation step diagram.

Each impact factor will not have the same degree of importance in relation to the object^17,18^, so the degree of impact and importance will be determined by weighting it. Hierarchical analysis is suitable for evaluating and analysing research objects affected by multiple factors^19,20^, and it is systematic and the calculations are clear, so it makes sense to use it to determine the weights of the impact factors^21,22^. The calculation steps of the hierarchical analysis method are shown in Fig. 7(b_ij_ is the importance of B_i_ to B_j_ with respect to the element A_n_ in the previous level).

#### Comprehensive metrics evaluation

Let u_k_ = u (a_i_ ∈ e_k_) be the degree to which each impact factor of the evaluation object at the k-th evaluation level, then the following equations can be obtained:

2 $$\mu k = \sum\limits_{i = 1}^{n} {\omega i\mu ik} \quad (i = 1, \, 2, \, \ldots , \, n;\quad \, k = 1, \, 2, \ldots , \, p)$$

3 $$\mu k = \left\{ {\mu_{1} , \, \mu_{2} , \, \ldots \, \mu_{p} } \right\}$$

Obviously, 0 ≤ u_k_ ≤ 1 and $${\sum }_{k=1}^{p}{\mu }_{k}=1$$。

### Creep level qualitative evaluating of crushed rock

#### Engineering geological data of the research object

**Figure 8:**
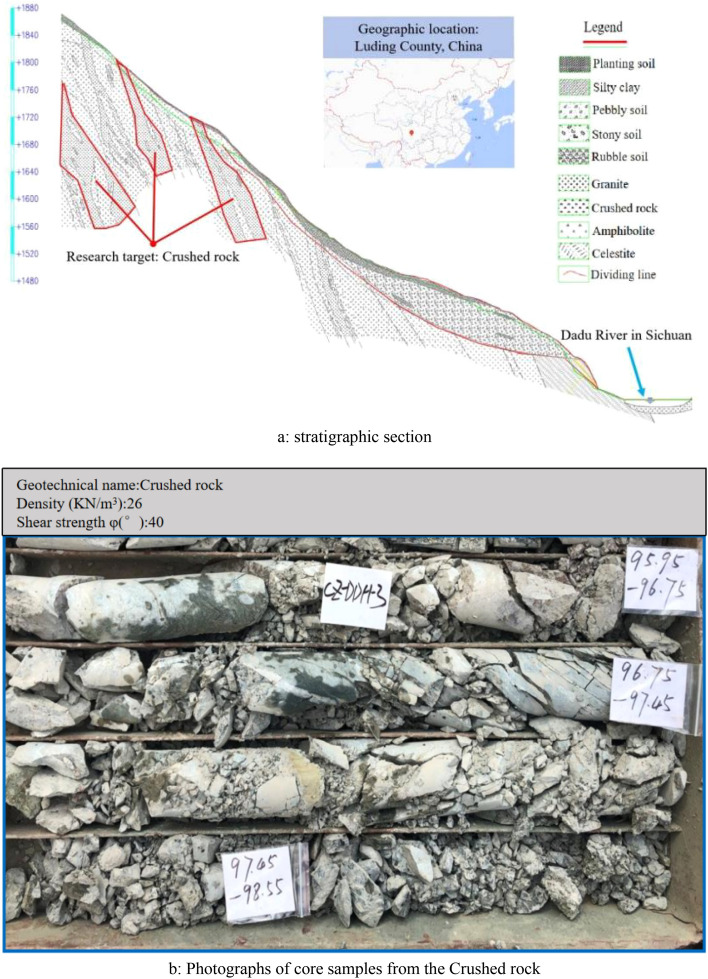
Engineering geological data of crushed rock.

This paper takes the crushed rock in a region in south-west China as the research object, the geological environment of this region is extremely complex, under the action of long-term high stress environment, the crushed rock inside the mountain is easy to occur creep deformation. According to the results of the ground investigation report, the study area is located in a tectonically mixed rock belt, and the area is mostly characterised by high and steep slopes^23^. The manuscript selected a typical high steep engineering slope in the region as the target^24^, according to the results of the rock coring in the deep layer of the slope, selected the crushed representative rock as the object of study, the relevant engineering geological data as shown in Fig. 8.

#### Establishing the qualitative evaluation model for the creep level of crushed rock

**Figure 9:**
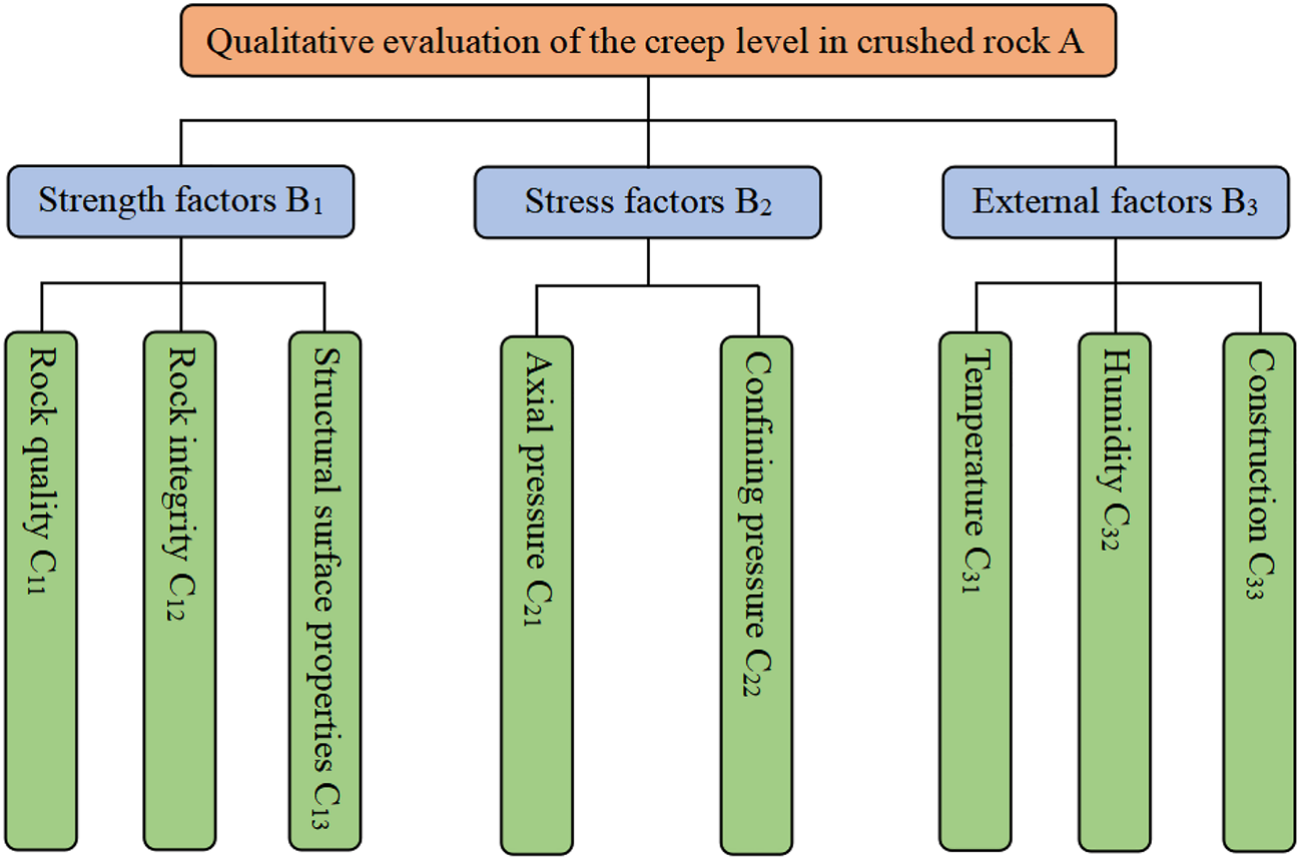
Creep level qualitative evaluation model of the crushed rock.

The paper has already carried out analysis of the 8 factors affecting the creep level of crushed rock, including rock quality, rock integrity, structural surface properties, axial pressure, confining pressure, temperature, humidity, construction. Among these, rock quality, rock integrity and structural plane properties characterise the strength of the rock itself, classifying them as strength factors; The axial pressure and confining pressure characterise the stress environment in which the rock is located and are classified as stress factors; Temperature, humidity, and construction are the external environments in which the rock mass is exposed, classifying them as external factors. Eventually, a creep level qualitative evaluation model of the crushed rock is formed with the creep level qualitative evaluation of the crushed rock as the target layer, the strength factor, stress factor, and external factor as the normative layer, and the eight specific influencing factors as the index layer. The model built is shown in Fig. 9.

#### Determining the weights

The AHP method was used to determine the weights of the indicators in the creep level qualitative evaluation model. Based on the processing of the ground investigation report and other related information of the research object, combined with the results of expert scoring, the comparison matrices A, B_1_, B_2_, B_3_ is constructed as shown below.

$$\begin{aligned} & A = \left( {\begin{array}{*{20}c} 1 & {\quad 3} & {\quad 4} \\ {1/3} & 1 & {\quad 3} \\ {1/4} & {\quad 1/3} & {\quad 1} \\ \end{array} } \right)\quad B1 = \left( {\begin{array}{*{20}c} 1 & {\quad 2} & {\quad 3} \\ {1/2} & {\quad 1} & {\quad 2} \\ {1/3} & {\quad 1/2} & {\quad 1} \\ \end{array} } \right) \\ & B2 = \left( {\begin{array}{*{20}c} 1 & {\quad 2} \\ {1/2} & {\quad 1} \\ \end{array} } \right)\quad B3 = \left( {\begin{array}{*{20}c} 1 & {\quad 2} & {\quad 4} \\ {1/2} & {\quad 1} & {\quad 2} \\ {1/4} & {\quad 1/2} & {\quad 1} \\ \end{array} } \right) \\ \end{aligned}$$

From the above comparison matrices, the weights of the normative layer layer to the target layer and each indicator layer to the normative layer can be calculated, and then checking the consistency of calculations. The results of the weighting calculations are shown in Table 2.

**Table 2:** Impact factor index weight.

Target layer	Normative layer	Weights	Index	Index layer weights
A	B_1_	0.608	C_11_	0.539
			C_12_	0.297
			C_13_	0.164
	B_2_	0.272	C_21_	0.667
			C_22_	0.333
	B_3_	0.120	C_31_	0.571
			C_32_	0.286
			C_33_	0.143

After obtaining the weights of the indicators at each level, the matrices were then tested for consistency. Firstly, the value of CI is calculated according to Eq. (4), and then it is compared with the random consistency index RI, which takes the values shown in Table 3.

4 $$CI = \frac{{\lambda_{\max } - p}}{p - 1}$$

**Table 3:** RI value table.

Order p	1	2	3	4	5	6	7	8	9
RI	0	0	0.58	0.9	1.21	1.24	1.32	1.41	1.45

Only when the random consensus ratio *CR* = *CI/RI* < *0.1* can the judgement matrix be established; Otherwise, the judgement matrix will have to be adjusted. The matrix consistency tests of the target layer to the criterion layer are as follows:

Matrix consistency test of the target layer against the normative layer:

$$\lambda_{{{\text{1max}}}} = {3}.0{7,}\,{\text{CI}} = 0.0{37,}\,{\text{RI}} = 0.{58,}\,{\text{CR}} = 0.0{64} < 0.{1}.$$

Matrix consistency test of the normative layer against the index layer:

$$\begin{aligned} & \lambda_{{{\text{2max}}}} = {3}.00{9,}\,{\text{CI}} = 0.00{5,}\,{\text{RI}} = 0.{58,}\,{\text{CR}} = 0.00{8} < 0.{1}. \\ & \lambda_{{{\text{3max}}}} = {1,}\,{\text{CI}} = 0.00,\,{\text{RI}} = 0. \\ & \lambda_{{{\text{4max}}}} = {3,}\,{\text{CI}} = 0,\,{\text{RI}} = 0. \\ \end{aligned}$$

From the above calculation results, it can be seen that each judgement matrix has passed the consistency test, so the constructed judgement matrix is valid and reasonable, and then the calculation can be continued to derive the weight vector:

$$\omega_{i} = \left[ {0.328,\, 0.181, \,0.100, \,0.181,\, 0.090, \,0.069,\, 0.034, \,0.017} \right]$$

#### Determining the unknown measurement matrix

In order to ensure the accuracy of the grading of each impact factor and to reduce errors in all aspects, the grading should be divided into as few grades as possible to reduce the influence of subjective factors. With reference to the domestic and foreign crushed rock creep theory researches, the creep level is divided into 4 levels. Respectively, Grade I: the creep level is weak, creep phenomenon is not evident; Grade II: the creep level is low, the deformation is small (0–10 mm); Grade III: the creep level is medium, the creep deformation is slightly large (1–5 cm); Grade IV: the creep level is high, the creep deformation is large (more than 5 cm). The division standard is shown in Table 4.

**Table 4:** Creep level classification criteria table.

Grade	Grade I	Grade II	Grade III	Grade IV
Creep deformation	Inconspicuous	0–10 mm	1–5 cm	> 5 cm
Score	90–100	75–89	60–74	0–59

By collating the measured data of the crushed rock in the study area; Surveying 100 people, including management, employees and experts responsible for the project area; Summarising the mean scoring table. The scoring results were obtained as shown in Table 5.

**Table 5:** Rating results.

Index	Grade I	Grade II	Grade III	Grade IV
Rock quality C_11_	22	36	30	12
Rock integrity C_12_	15	35	35	15
Structural surface properties C_13_	20	30	32	18
Axial pressure C_21_	25	40	25	10
Confining pressure C_22_	25	40	30	5
Temperature C_31_	35	30	25	10
Humidity C_32_	22	38	26	14
Construction C_33_	30	40	20	10

According to Eq. (1) Uncertainty Measurement Matrix Calculation Method can get the Uncertainty Measurement Matrix, as shown in Eq. (5).

5 $$\mu_{ij} = \left[ {\begin{array}{*{20}c} {0.22} & {\quad 0.36} & {\quad 0.30} & {\quad 0.12} \\ {0.15} & {\quad 0.35} & {\quad 0.35} & {\quad 0.15} \\ {0.20} & {\quad 0.30} & {\quad 0.32} & {\quad 0.18} \\ {0.25} & {\quad 0.40} & {\quad 0.25} & {\quad 0.10} \\ {0.25} & {\quad 0.40} & {\quad 0.30} & {\quad 0.05} \\ {0.35} & {\quad 0.30} & {\quad 0.25} & {\quad 0.10} \\ {0.22} & {\quad 0.38} & {\quad 0.26} & {\quad 0.14} \\ {0.30} & {\quad 0.40} & {\quad 0.20} & {\quad 0.10} \\ \end{array} } \right]$$

Combined with the results of the weighting calculations, the final composite measure evaluation vector is calculated:

6 $$\mu = \omega_{t} \mu_{tj} = \left[ {\begin{array}{*{20}c} {0.224} & {\quad 0.360} & {\quad 0.295} & {\quad 0.121} \\ \end{array} } \right]$$

According to the calculation theory of the unconfirmed measure model, the maximum value in the final derived comprehensive measure evaluation vector μ is 0.360, and its corresponding qualitative evaluation grade of the creep level is Class II, indicating that the creep level of the crushed rock within the mountain in the region is low, and the amount of creep deformation is small. And from the calculation process, it can be seen that, among the eight indicators affecting the creep level of the research object, the results leading to the low creep level should be the result of the combined coupling of four influencing factors: rock quality, rock integrity, axial pressure, and confining pressure, other factors have a low effect on creep.

Firstly, it can be found from the previous engineering geological data (core sampling diagram) that the crushed rock is extremely fragmented and low rock integrity, which will make it susceptible to creep even lead to a large creep deformation. However, the crushed rock properties are good (the rock has a gravity of 26 kn/m^3^ and an internal friction angle of 40°, which indicates that the strength of the crushed rock is high). It is assumed that its ability to resist creep deformation is strong. In addition, the stratigraphical sections show that the maximum burial depths of the crushed rock is up to 300 m, as a result, the crushed rock is in a good stress environment in the stratigraphy. Which means that the crushed rock is stabilised, and has a high resistance to creep deformation caused by other factors (such as construction, temperature and humidity, et al.)

Therefore, under the effect of mutual coupling of various factors, the final result is that the creep level of the crushed rock in the study area is low, and the amount of deformation is small (the creep level of the crushed rock is evaluated to be class II).

## Concluding

Taking the crushed rock in Southwest China as a research object, the paper systematically analysed eight factors affecting the creep of the rock. Then based on the theory of unconfirmed measurement and hierarchical analysis, a model was established to qualitatively evaluate the creep level of the crushed rock. Finally, the established model was used to qualitatively evaluate the creep level of the crushed rock in the study area. It is concluded that the creep level of the crushed rock is evaluated at Grade II, which is characterised by a low creep level of the rock and a small amount of deformation (0–10 mm). The research results can provide a reference for the creep analysis of crushed rock and provide a basis for the safe construction of engineering slopes.

## Data Availability

The data used to support the findings of this study are available from the corresponding author upon request.

## References

[1] Shuxin Y, Rui Y, Xiaofeng C (2012). Characterisation of measured stresses in the Chinese mainland, active land masses and north-south seismic zones. Geophys. J..

[2] Zhiyong, W. *Patterns of Geological Hazards and Disaster-Causing Mechanisms in the Mountainous Mining Collapse Zone of Northern Hebei* (China University of Mining and Technology, 2022).

[3] Ministry of Construction of the People’s Republic of China (2009). Specification for Geotechnical Investigation: GB50021-2009.

[4] Wu K, He M, Yuan Z, Liu X, Luo B, Ma X, Ma C (2024). Characterizing rock transverse anisotropic spatial variations using digital drilling. Geoenergy Sci. Eng..

[5] Yuanfang C (2015). Rock Mechanics for Oil and Gas Engineering.

[6] Yao W (2021). Mechanical Characteristics of Creep Damage in Saturated Frozen Sandstone of Western Cretaceous.

[7] Yang B, He M, Xiao Z (2023). Effect of horizontal stress on fractal characteristics of rockburst fragments in coal mining. Energy.

[8] He M, Ding M, Yuan Z (2023). Numerical simulation of rock bursts triggered by blasting disturbance for deep-buried tunnels in jointed rock masses. Comput. Geotech..

[9] Zhang R, Wu S, Xie C (2021). Study on the geological condition analysis and grade division of high altitude and cold stope slope. Sustainability.

[10] Raoufi M, RobinsonFayek A (2018). Fuzzy agent-based modeling of construction crew motivation and performance. J. Comput. Civ. Eng..

[11] Guofang C, Hao W (2021). Tunnel gas risk evaluation based on EW-AHP and unconfirmed measure theory. Non-ferrous Met. Sci. Eng..

[12] Li JL, Yuan C, Zhang B, Sui B (2021). Evaluation and application of surrounding rock stability based on the improved weighting multidimensional cloud model. Adv. Civ. Eng..

[13] Long GY, Wang H, Hu K (2024). Probability prediction method for rockburst intensity based on rough set and multidimensional cloud model uncertainty reasoning. Environ. Earth Sci..

[14] Gao Y, Gao F, Zhou K (2020). Evaluation model of surrounding rock stability based on fuzzy rock engineering systems (RES)-connection cloud. Bull. Eng. Geol. Environ..

[15] Yang Y, Huang G, Meng L (2021). Surrounding rock stability classification method of coal roadway based on in situ stress. Adv. Civ. Eng..

[16] Shi XY, Fan JQ, Guo P (2021). Evaluation of the large deformation grade cloud model of surrounding rock based on combination weighting method. IOP Conf. Ser. Earth Environ. Sci..

[17] Dao DV, Bui QAT, Nguyen DD (2022). Prediction of interlayer shear strength of double-layer asphalt using novel hybrid artificial intelligence models of ANFIS and metaheuristic optimizations. Constr. Build. Mater..

[18] Nguyen LH, Vu DQ, Nguyen DD (2023). Prediction of falling weight deflectometer parameters using hybrid model of genetic algorithm and adaptive neuro-fuzzy inference system. Front. Struct. Civ. Eng..

[19] Yin Z, Zhang Q, Laouafa F (2023). Multiscale multiphysics modeling in geotechnical engineering. J. Zhejiang Univ. Engl. Ed. Ser. A Appl. Phys. Eng..

[20] Wang ZY, Li YW, Wu ZJ (2023). Hierarchical scaling model for size effect on tensile strength of polycrystalline rock. Int. J. Mech. Sci..

[21] Egbueri JC, Khan MYA (2023). Understanding the geotechnical and geomechanical characteristics of erodible soils: A study incorporating soft computational modeling techniques. Environ. Dev. Sustain..

[22] Yu G, Lou Y, Dong H (2023). A multilevel hierarchical parallel algorithm for large-scale finite element modal analysis. CMC-Comput. Mater. Contin..

[23] Tingjun C, Shiguo X, Qiang C (2019). Long-term deformation and stability analysis of gravity-anchored slopes on the Kangding bank of the Luding Dadu River Bridge. J. Eng. Geol..

[24] Wei K, Xia Li, Qiang W (2021). Research on bridge design of Changdu to Linzhi section of Sichuan-Tibet railway. Railw. Stand. Des..

